# Pharmacokinetics of Tedizolid and Pseudoephedrine Administered Alone or in Combination in Healthy Volunteers

**DOI:** 10.3390/jcm7060150

**Published:** 2018-06-14

**Authors:** Shawn Flanagan, Sonia L. Minassian, Philippe Prokocimer

**Affiliations:** 1 Merck & Co., Inc., Kenilworth, NJ 07033, USA; ppkprokocimer@gmail.com; 2 Minassian Biostatistics, Inc., San Diego, CA 92121, USA; sminassi@yahoo.com

**Keywords:** antibiotics, drug interactions, oxazolidinone, infectious disease, acute bacterial skin infections, pharmacokinetics

## Abstract

Therapeutic doses of tedizolid phosphate, an oxazolidinone antibiotic, lack monoamine oxidase inhibition in vivo, potentially resulting in an improved safety profile versus other oxazolidinones. This randomized, double-blind, placebo-controlled, 2-period, 2-sequence, crossover, phase 1 study (NCT01577459) assessed the potential for pharmacokinetic (PK) interactions between tedizolid and pseudoephedrine. Eighteen healthy volunteers (age: 18–45 years) were block-randomized to 1 of 2 treatment sequences containing 2 treatment periods (tedizolid phosphate or placebo once daily for 4 days; single dose of pseudoephedrine 60 mg on day 5) separated by a 2-day washout. Median time to maximum plasma concentration for tedizolid and pseudoephedrine ranged from 3 to 4 h, regardless of treatment coadministration. Steady-state tedizolid had no effect on the PK of pseudoephedrine; geometric mean ratio and 90% confidence interval remained within the no-effect 0.8 to 1.25 boundaries. The maximum observed concentration of tedizolid decreased by approximately 14% when pseudoephedrine was coadministered; no changes in the area under the plasma concentration-time curve or the minimum observed plasma concentration occurred. All adverse events (AEs) were mild, and there were no serious AEs or study drug discontinuations. No meaningful PK interactions occurred between tedizolid and pseudoephedrine, and tedizolid was well tolerated when administered in conjunction with pseudoephedrine.

## 1. Introduction

Agents approved for the treatment of acute bacterial skin and skin structure infections (ABSSSI) are generally efficacious, but toxicity and microbial resistance may limit their use [1,2]. Given the increasing prevalence of multidrug-resistant, gram-positive pathogens, there is a need for new and effective antibiotics with proven antibacterial activity [2,3]. Oxazolidinones are one such class of antibacterial agents.

The oxazolidinone antibiotic linezolid, an inhibitor of monoamine oxidase (MAO), is approved for the treatment of uncomplicated and complicated skin and skin structure infections; however, significant safety concerns have emerged since its approval [4]. Coadministration of linezolid with other MAO inhibitors, adrenergic drugs, or serotonergic drugs is restricted because it may produce cardiovascular (e.g., hypertension) or neurologic (i.e., serotonin syndrome) treatment-emergent adverse events (AEs) that can be fatal in some patients [3,4]. The potential severity of these complications restricts investigation of the interaction in patients for ethical reasons; preclinical and healthy volunteer (phase 1) studies are used to better control the experimental parameters. Additionally, clinical studies have demonstrated that linezolid increases blood pressure in patients who have uncontrolled hypertension and in those who are taking sympathomimetic drugs such as pseudoephedrine [4,5], which are associated with increases in systolic blood pressure (SBP) and heart rate (HR) [6].

Tedizolid phosphate, a novel oxazolidinone antibiotic approved for the treatment of ABSSSI (200 mg once daily for 6 days) [7,8], demonstrated noninferiority compared with linezolid (600 mg twice daily for 10 days) in 2 randomized controlled phase 3 clinical trials (ESTABLISH-1 and ESTABLISH-2) [9,10]. Tedizolid, like linezolid, is a reversible inhibitor of MAO type A (MAO-A) and type B (MAO-B) in vitro [7,8]. Unlike linezolid, however, therapeutic doses of tedizolid phosphate lack MAO inhibition in vivo and subsequent hypertensive adrenergic or serotonergic adverse consequences, as reported in nonclinical and clinical studies in healthy volunteers [11].

This report presents the pharmacokinetic (PK) data that were generated during the phase 1 study (NCT01577459). The pharmacodynamic data (PD) from this same study were previously reported and showed a lack of PD interaction between tedizolid phosphate and pseudoephedrine, in that no significant difference was detected in the maximum change in SBP of healthy volunteers who either received pseudoephedrine with tedizolid phosphate compared with those who received pseudoephedrine with placebo (11.6 mmHg vs. 12.1 mmHg; *p =* 0.73) [11]. Specifically, only 4 (22%) of the participants receiving pseudoephedrine with tedizolid phosphate experienced an increase in SBP of ≥15 mmHg, while 5 (28%) of the participants who received pseudoephedrine with placebo experienced the same SBP increase. Similarly, no significant difference was found between the maximum HR changes in the group receiving pseudoephedrine with tedizolid phosphate compared with those who received pseudoephedrine with placebo (13.6 beats/min vs. 15.2 beats/min, respectively; *p =* 0.17).

To ensure that no underlying PK differences confounded the previously reported PD assessment, here we assess the potential for PK interactions between tedizolid phosphate and pseudoephedrine [11]. As a single agent, the PK of tedizolid phosphate has been characterized [7]. The prodrug, tedizolid phosphate, is rapidly cleaved into the active moiety, tedizolid following oral and intravenous administration, and has a half-life of approximately 12 h. Steady-state concentrations of tedizolid are reached in approximately 3 days and tedizolid accumulation is approximately 30%. In plasma, tedizolid accounts for 95% of the total radiocarbon AUC and there are no other circulating metabolites. The majority of the dose is metabolized to an inactive sulfate conjugate and is eliminated via the liver with approximately 82% appearing in the feces and 18% in urine [12]. The PK profile of pseudoephedrine as a single agent has also been documented. The half-life of pseudoephedrine is approximately 5–8 h [13]. Steady plasma levels are achieved after 48 h [14]. Pseudoephedrine is primarily eliminated unchanged in the urine with small amounts of a hepatic metabolite. While minimal or no PK interaction between tedizolid and pseudoephedrine would be expected, we sought to confirm the absence of a yet unknown mechanism.

## 2. Experimental Section

### 2.1. Ethics

This study (ClinicalTrials.gov registration number NCT01577459) was conducted by Vince and Associates Clinical Research (Overland Park, KS, USA) in accordance with the ethical principles that have their origin in the Declaration of Helsinki and are consistent with Good Clinical Practice and with applicable national and institutional regulatory requirements. The study protocol (sponsor reference TR701-114) was approved by the MidLands Independent Review Board (Overland Park, KS, USA), and all participants provided written informed consent before any study procedures were performed.

### 2.2. Study Design and Treatments

This randomized, double-blind, placebo-controlled, 2-period, 2-sequence, crossover, phase 1 study was designed to evaluate the effects of steady state 200 mg once-daily oral tedizolid phosphate or placebo on single-dose PK of pseudoephedrine in healthy participants. Steady-state PK of tedizolid, the biologically active moiety of tedizolid phosphate, with and without pseudoephedrine administration, was also determined.

Participants (*N* = 18) were block-randomized to 1 of 2 treatment sequences, with each sequence containing 2 treatment periods separated by a 2-day washout. During each treatment period, tedizolid phosphate or placebo was administered once daily for 4 days to reach steady state. On day 5 of each treatment period, a single dose of immediate-release pseudoephedrine 60 mg was administered simultaneously with the study drug.

### 2.3. Participants

Participants included male and female healthy volunteers aged 18 to 45 years whose body mass index (BMI) ranged from ≥19.0 kg/m^2^ to ≤31.0 kg/m^2^, and met the following criteria: no clinically significant abnormalities identified by a detailed medical history, complete physical examination, 12-lead electrocardiograms and clinical laboratory tests, and negative pregnancy test results at screening and on study day −1 in females of childbearing potential.

Exclusion criteria were clinically significant history or evidence of cardiovascular, respiratory, hepatic, renal, gastrointestinal, endocrine, neurologic, immunologic, or psychiatric disorder(s); history of alcohol or substance abuse; SBP >130 mmHg or <90 mmHg; diastolic blood pressure (DBP) >90 mmHg or <60 mmHg; HR >90 bpm or <50 bpm after 10 min supine at screening and on study day −1; and QT interval corrected for HR using Fridericia’s formula >500 ms. Previous or concomitant use of prescription or nonprescription drugs, herbal supplements, illicit drugs, or tobacco (≥10 cigarettes/day) or nicotine-containing products was prohibited during the study. Diets high in tyramine or containing alcohol, grapefruit, caffeine, or xanthine were also prohibited. Previous participants in a tedizolid phosphate or tedizolid clinical study were excluded.

### 2.4. Measurements

Blood samples were collected at 0, 1, 2, 3, 4, 6, 8, 12, and 24 h after study drug administration on study days 4 and 5. The PK of tedizolid, assessed on day 4, was compared with the PK of tedizolid assessed on day 5 with the coadministration of pseudoephedrine. Pseudoephedrine PK on day 5 was compared between treatment periods (administration with tedizolid phosphate or placebo).

Throughout the study, safety assessments were collected, and these included vital signs, AEs, clinical laboratory evaluations, and physical examinations.

Participants were discharged from the clinic on study day 13, and follow-up assessments were conducted 7 days (±1 day) after discharge.

### 2.5. Statistical Analyses

The intent-to-treat (ITT) analysis set included all participants who gave informed consent and were randomly assigned to a treatment sequence. The PK analysis set included participants who received at least 1 administration of pseudoephedrine or tedizolid phosphate and had at least 1 postadministration collection of blood. The safety analysis set included participants who received any study drug.

Participant disposition, baseline demographics, and safety outcomes were summarized using descriptive characteristics. PK parameters evaluated included area under the plasma concentration-time curve from hour 0 to the last quantifiable time point (AUC_0–t_), AUC_0–24_, AUC_0–∞_, maximum observed concentration (C_max_), minimum observed concentration (C_min_) for tedizolid only, and time to maximum observed concentration (T_max_). Mean PK values were compared between the 2 treatments using analysis of variance (ANOVA) models with the log of the PK parameter as the dependent variable, treatment as a fixed factor, and participant as a random factor. Results from the ANOVA model were used to generate geometric mean (GM) values to calculate a GM ratio (GMR) for each parameter, and a 90% confidence interval (CI) for each GMR.

## 3. Results

### 3.1. Baseline Characteristics

All participants met the criteria for all analysis sets, and thus, the ITT, PK, and safety analysis sets were identical and included data from all 18 randomly assigned participants (9 per treatment sequence). Most (83.3%) participants were men, 50% were white, and 44.4% were black; their mean (±standard deviation) age was 34.4 (±7.5) years, and their mean BMI was 26.7 (±2.5) kg/m^2^.

### 3.2. Pharmacokinetics

**Figure 1:**
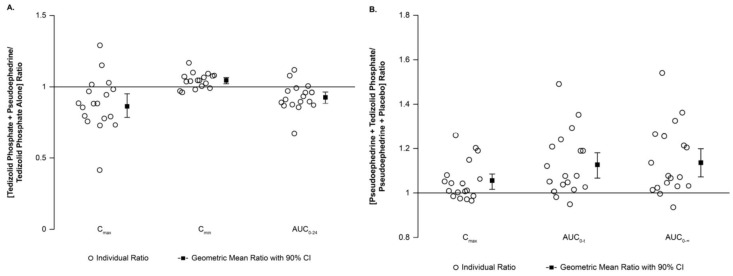
By-participant display of PK parameter ratios. (**A**) Individual tedizolid C_max_, C_min_, and AUC_0__–24_ treatment ratios (tedizolid + pseudoephedrine/tedizolid alone), GMRs, and corresponding 90% CIs; (**B**) Individual pseudoephedrine C_max_, AUC_0__–t_, and AUC_0__–∞_ treatment ratios (pseudoephedrine + tedizolid/pseudoephedrine + placebo), GMRs, and corresponding 90% CIs. **Abbreviations:** AUC_0__–t_, area under the plasma concentration-time curve from hour 0 to last quantifiable time point; AUC_0–24_, area under the plasma concentration-time curve from hour 0 to hour 24; AUC_0__–∞_, area under the plasma concentration-time curve from hour 0 extrapolated to infinity based on the apparent terminal rate constant; CI, confidence interval; C_max_, maximum observed concentration; C_min_, minimum observed concentration; GM, geometric mean; GMR, geometric mean ratio; PK, pharmacokinetic.

Median T_max_ values for tedizolid and pseudoephedrine were similar (3 to 4 h) regardless of coadministration (data not shown). Steady state tedizolid had no effect on the PK of pseudoephedrine; the GMR and 90% CIs remained within the no-effect 0.8 to 1.25 boundaries (Table 1) [15]. C_max_ of tedizolid decreased by approximately 14% when pseudoephedrine was coadministered; no changes in AUC or C_min_ were observed, as indicated by the GMR and 90% CIs remaining between 0.8 and 1.25 (Table 1). Most participants had similar tedizolid PK exposure with and without pseudoephedrine (Figure 1A) and similar pseudoephedrine exposure with and without tedizolid phosphate (Figure 1B).

### 3.3. Safety

Overall, tedizolid phosphate 200 mg for 5 days plus pseudoephedrine on study day 5 was well tolerated. Similar rates of AEs were reported during the placebo treatment period (*N* = 5) and the tedizolid phosphate treatment period (*N* = 4), and all AEs occurred after the first administration of pseudoephedrine (data not shown). All AEs were mild, none were serious, and no participant discontinued the study drug. Only headache and blood bilirubin increase were reported for more than 1 participant. Headaches occurred in 1 participant receiving placebo and 1 participant receiving tedizolid phosphate. Elevated bilirubin was found in 1 participant during both treatment periods and 1 participant only during treatment with tedizolid phosphate. Throughout the study, no notable laboratory or physical findings were observed.

Common AEs associated with pseudoephedrine include dry mouth, nausea, dizziness, insomnia, and nervousness [13]. Common AEs associated with tedizolid include nausea, headache, diarrhea, vomiting, and dizziness [7].

## 4. Discussion

These phase 1 study results demonstrated that there was no PK drug–drug interaction between tedizolid and pseudoephedrine. The PK of pseudoephedrine was unchanged when it was coadministered with tedizolid phosphate at steady state. No meaningful changes in tedizolid PK were noted with pseudoephedrine coadministration: C_max_ showed a slight decrease (14%) when pseudoephedrine was coadministered, whereas AUC values (the key determinant for efficacy) and C_min_ (related to safety) were unchanged. Plasma concentrations of tedizolid and pseudoephedrine each reached C_max_ at approximately the same time. Hence, the lack of pharmacologic interaction with coadministration cannot be explained by the drug administration scheduling.

These data confirm previously published results demonstrating that in various experimental settings, reflecting adrenergic and serotonergic systems, there were no MAO-mediated PD interactions between tedizolid and pseudoephedrine [11]. In contrast to the increase in SBP and DBP observed with linezolid [5], coadministration of tedizolid phosphate and pseudoephedrine did not significantly affect SBP, DBP, or HR [11]. The lack of MAO inhibition observed with tedizolid phosphate in vivo suggests an improved safety profile compared with linezolid, presenting a meaningful advantage in the many patients taking adrenergic or serotonergic agents concomitantly. A plausible explanation for the lack of in vivo drug–drug interaction is that free tedizolid C_max_ concentrations are consistently well below the tedizolid half-maximal inhibitory concentration (IC_50_) for MAO (at 20% of MAO IC_50_) inhibition, a standard marker of pharmacologic activity.

Although generally well tolerated as a class, prolonged use of oxazolidinones, particularly linezolid, has been associated with reversible myelosuppression, specifically thrombocytopenia [4]. An assessment of 2 phase 3 clinical trials (ESTABLISH-1 and ESTABLISH-2) that compared tedizolid with linezolid in the treatment of patients with acute bacterial skin and skin structure infections (ABSSSIs) showed that 6 days of 200 mg once daily tedizolid was associated with a lower incidence of thrombocytopenia versus 10 days of 600 mg linezolid twice daily (3.2% and 5.6%, respectively) [16]. The ESTABLISH-1 and ESTABLISH-2 trials confirmed this tedizolid dosing regimen as non-inferior to the linezolid regimen, which was both longer and uses a higher drug concentration [9,10]. In vitro susceptibility studies demonstrate that the minimum inhibitory concentration (MIC) of tedizolid is lower than that of linezolid for *Staphylococcus aureus* (MIC_90_ of 0.25 µg/mL vs. 2 µg/mL), coagulase-negative staphylococci (MIC_90_ of 0.25 µg/mL vs. 2 µg/mL), as well as several other isolates including viridans group streptococci and beta-hemolytic streptococci [17]. The difference in concentration and duration of therapeutic dose of tedizolid and linezolid may contribute to this and other findings that suggest tedizolid has a favorable safety profile while offering non-inferior efficacy compared with linezolid in patients with ABSSSIs [9,10,11,16,18].

## 5. Conclusions

In conclusion, these results demonstrate that no meaningful PK interactions occur between tedizolid and pseudoephedrine, supporting previously published results on lack of an MAO PD interaction. Tedizolid was well tolerated when administered with pseudoephedrine.

## Figures and Tables

**Table 1:** Effects on plasma PK parameters (PK analysis set).

Parameter	GM ^a^		GMR	
Effect of tedizolid on pseudoephedrine				
	Pseudoephedrine + tedizolid *N* = 18	Pseudoephedrine + placebo *N* = 18	Pseudoephedrine + tedizolid/pseudoephedrine + placebo	90% CI ^b^
C_max_ (ng/mL)	210.2	200.2	1.050	1.016–1.084
AUC_0–t_ (ng·h/mL)	2227.2	1986.1	1.121	1.065–1.180
AUC_0–∞_ (ng·h/mL)	2422.6	2138.9	1.133	1.071–1.198
Effect of pseudoephedrine on tedizolid				
	Tedizolid + pseudoephedrine *N* = 18	Tedizolid + placebo *N* = 18	Tedizolid + pseudoephedrine/tedizolid + placebo	90% CI ^b^
C_max_ (ng/mL)	1855.8	2157.5	0.860	0.780–0.948
C_min_ (ng/mL)	417.3	401.3	1.040	1.019–1.061
AUC_0–t_ (ng·h/mL)	22,690.9	24,578.5	0.923	0.883–0.965
AUC_0–24_ (ng·h/mL)	22,688.4	24,598.2	0.922	0.882–0.964

^a^ Least squares mean from ANOVA model, calculated by transforming the natural log mean back to the linear scale (i.e., geometric mean). The ANOVA model included the log of the PK parameter as the response, treatment as a fixed factor, and participant as a random factor; ^b^ 90% CI for the GMR. **Abbreviations:** ANOVA, analysis of variance; AUC_0__–t_, area under the plasma concentration-time curve from hour 0 to last quantifiable time point; AUC_0–24_, area under the plasma concentration-time curve from hour 0 to hour 24; AUC_0__–∞_, area under the plasma concentration-time curve from hour 0 extrapolated to infinity based on the apparent terminal rate constant; CI, confidence interval; C_max_, maximum observed concentration; C_min_, minimum observed concentration; GM, geometric mean; GMR, geometric mean ratio; PK, pharmacokinetic.
